# Fate bias during neural regeneration adjusts dynamically without recapitulating developmental fate progression

**DOI:** 10.1186/s13064-017-0089-y

**Published:** 2017-07-13

**Authors:** Jeremy Ng Chi Kei, Peter David Currie, Patricia Regina Jusuf

**Affiliations:** 1 Australian Regenerative Medicine Institute, Monash University, Clayton, VIC 3800 Australia; 2 School of Biosciences, University of Melbourne, Parkville, VIC 3010 Australia

**Keywords:** Neural regeneration, Zebrafish, Fate bias, Retina, Fate specification

## Abstract

**Background:**

Regeneration of neurons in the central nervous system is poor in humans. In other vertebrates neural regeneration does occur efficiently and involves reactivation of developmental processes. Within the neural retina of zebrafish, Müller glia are the main stem cell source and are capable of generating progenitors to replace lost neurons after injury. However, it remains largely unknown to what extent Müller glia and neuron differentiation mirror development.

**Methods:**

Following neural ablation in the zebrafish retina, dividing cells were tracked using a prolonged labelling technique. We investigated to what extent extrinsic feedback influences fate choices in two injury models, and whether fate specification follows the histogenic order observed in development.

**Results:**

By comparing two injury paradigms that affect different subpopulations of neurons, we found a dynamic adaptability of fate choices during regeneration. Both injuries followed a similar time course of cell death, and activated Müller glia proliferation. However, these newly generated cells were initially biased towards replacing specifically the ablated cell types, and subsequently generating all cell types as the appropriate neuron proportions became re-established. This dynamic behaviour has implications for shaping regenerative processes and ensuring restoration of appropriate proportions of neuron types regardless of injury or cell type lost.

**Conclusions:**

Our findings suggest that regenerative fate processes are more flexible than development processes. Compared to development fate specification we observed a disruption in stereotypical birth order of neurons during regeneration Understanding such feedback systems can allow us to direct regenerative fate specification in injury and diseases to regenerate specific neuron types in vivo.

## Background

All vertebrates show some potential for neural regeneration in the central nervous system, including the retina. In lower vertebrates, such as zebrafish, the adult retina contains multiple neurogenic cell sources including progenitors in the ciliary margin zone, and Müller glia [1–6]. Retinal injuries activate Müller glia to de-differentiate and reactivate neurodevelopmental gene expression cascades in zebrafish [7–13]. Although the regenerative response is more limited in mammals, glia activation and proliferation has also been observed in rodent [14], and human in vitro studies [15, 16], additional to chick [17] and amphibian (reviewed in [18]). Fate specification during development is controlled primarily by intrinsic gene expression, but also influenced by environmental cues [19–25]. However, little is known about the extent to which regenerating adult progenitors may utilise such cues and whether conserved developmental processes are recapitulated.

Efficient glial driven functional visual recovery [11, 13, 26–30] and regeneration of ablated photoreceptor, ganglion or bipolar cells [26, 31–34] occurs in zebrafish. Proliferative cells show a bias towards generating ablated cell fates, but also generate non-ablated cells [33, 35, 36]. This shows their intrinsic multipotency and may reflect recapitulation of intrinsic molecular processes that control temporal cell fate decision as observed in development. Assessing these questions will have profound implications for targeted and efficient regeneration within the tissue to direct regenerative processes including fate choices, differentiation and circuit integration.

Our study has used extended time-course labelling to mark all newly regenerated cells and quantified the proportion of each retinal neuron type regenerated (i.e. ablated vs. non-ablated). Differential neural cell ablation was found to direct fate specification in regenerating progenitors dynamically. In contrast to previous studies, we identify a key early time point at which ablated neurons are almost exclusively regenerated. Subsequently, such cell specific regeneration restores the appropriate neural proportions, and progenitors switch towards an unbiased mode. Unexpectedly and not previously described, our results show a lack of conservation in the developmental histogenic order during regeneration. Thus, regenerating progenitors display a remarkable adaptability by using extrinsic feedback to dynamically adjust fate specification. This correcting of neural composition might aid with appropriate synaptic circuit formation and visual function recovery in vivo.

## Methods

### Zebrafish husbandry

Zebrafish (*Danio rerio*) of either gender were maintained at FishCore at Monash University or Walter and Eliza Hall Institute of Medical research zebrafish facility in accordance with local animal guidelines. Animals were assigned to the various experimental groups randomly and no animals were excluded from analysis. Fishlines used include TU, Tg(*ptf1a:Gal4*) kindly provided by Prof. Leach [37], Tg*(UAS:nfsb-mCherry*) [38], a gift from Prof. Lieschke, Tg(*gfap:GFP*) generated by Dr. Bernardos and Prof. Raymond [39], Tg(*vsx1:GFP*) provided by Prof. Higashijima [40], Tg(*atoh7:GFP*) generated by Drs Zolessi and Poggi [41]. Lines were crossed to generate double and triple transgenic lines such as Tg(*atoh7:GFP/ptf1a:Gal4/UAS:nfsb-mCherry*) and Tg(*vsx1:GFP/ptf1a:Gal4/UAS:nfsb-mCherry*). Juveniles were maintained according to standard protocol, staged as previously described [42], and used before and after free feeding stages.

### Mechanical ablation (needle stick injury)

One week old zebrafish were anaesthetised in 0.0006% tricaine methanesulfonate and placed on 2% low melt agarose coated petri dishes. Retinal injury was performed using glass needles, pulled from a 1.0 mm O.D × 0.78 mm I.D glass capillary (Harvard Apparatus, Holliston, MA, USA). Injury was conducted at 6 different locations on the eye. The zebrafish were recovered in fresh E3 solution and subsequently monitored for welfare purposes.

### Genetic ablation (metronidazole treated nitroreductase injury)

One week old Tg(*ptf1a:Gal4/UAS:nfsb-mCherry*) zebrafish were incubated in 10 mM metronidazole/0.2% DMSO in E3 (NaCl, KCl, CaCl_2_.2H_2_O, MgCl_2_.6H_2_0, methylene blue) solution for 8 h at 28 °C. Zebrafish were rinsed 3 times in fresh E3 media, and monitored for welfare purposes.

### 5-bromo-2′-deoxyuridine (BrdU) exposure

The proliferative phase and fate tracking of newly generated cells was performed using BrdU incorporation. Larvae were swum in 2 mM BrdU diluted in E3 (pH 7.0). Larvae were swum for 24 h to BrdU at stages 0 to 7 days post injury (dpi). For prolonged BrdU pulse experiments, larvae were swum overnight for 16 h every day from 3 dpi to 7 dpi, and recovered in fresh E3 for 8 h during the day.

### Immunohistochemistry

Larvae were fixed in 4% paraformaldehyde (PFA) in phosphate buffered saline (PBS, pH 7.4), cryoprotected in 7.5% gelatine (GL005/500G, Science Supply Australia, Mitcham, Australia) / 15% sucrose in PBS solution, and cryostat sectioned at 14 μm thickness using a Leica CM3050S Cryostat. Antibody staining was performed at room temperature using standard protocols. Sections were blocked in 5% fetal bovine serum (FBS)/0.5% Triton x-100 in PBS, and incubated overnight in primary antibody diluted in the same block solution. Secondary antibodies used (all 1:400 from Thermo Fisher Scientific, Mulgrave, Australia) were anti-mouse Alexa Fluor-488 (cat. number A11001) or Alexa Fluor-546 (cat. number 1256168), anti-rabbit Alexa Fluor-546 (cat. number A11010) and anti-sheep Alexa Fluor-546 (cat. number A21098) diluted in the same block solution. Nuclei were counterstained with 4′,6-diamidino-2-phenylindole (DAPI, cat. number D9542-10MG, Sigma-Aldrich, Castle Hill, Australia) and sections mounted in Mowiol (cat. number 81381-250G, Sigma-Aldrich, Castle Hill, Australia).

### Antibodies

Detection of proliferating cells was performed with mouse anti-BrdU (1:500, Sigma Aldrich, cat. number 11170376001, clone BMC9318) [19], which specifically labels BrdU [43].

Characterisation of cell death resulting from each injury paradigm was detected using the terminal deoxynucleotidyl transferase dUTP nick end labeling (TUNEL) with the in situ cell death detection kit, fluorescein including sheep anti-fluorescein Fab fragment antibody (1:500, Sigma Aldrich, cat. number 11684795910, Castle Hill, Australia).

Proliferating cells were detected with rabbit anti-PCNA (proliferating cell nuclear antigen) antibody (1:500, Sigma Aldrich, cat. number SAB2701819), which specifically recognises zebrafish PCNA (manufacturer’s information).

### Image acquisition

Images of fixed sections were obtained on a Zeiss Z1 (20× objective) using an AxioCam (HRm 13-megapixel, monochrome) with Apotome and Axiovision software. Brightness and contrast were adjusted with Photoshop (Adobe, San Jose, CA, USA).

### Analysis

The number of larvae analysed is indicated in the figure legends. Co-labelling quantification was performed only on images taken with the Apotome to provide single optical sections similar to confocal images. For cell death, cell cycle exit, cell fate specification, TUNEL, PCNA, Atoh7:GFP and Vsx1:GFP co-labelled cell were analysed across the central retina, excluding the ciliary margin zone (a region of developmental neurogenesis) and standardized to 400 μm, which represents the width of the layers in an average retinal section. Because the section thickness is the same for all experiments, all quantifications are directly comparable. All results are presented as mean ± SEM. The relative proportion of ablated cell types regenerated at each time point was compared to the control uninjured proportions using student’s *t*-test.

## Results

### Cell death in distinct neural populations can be efficiently targeted by specificity of injury

In zebrafish, after the initial developmental wave (first 72 h postfertilisation (hpf) [44, 45]), growth via developmental neurogenesis continues in the very peripheral edge in a specialised niche termed the ciliary margin zone (CMZ) [reviewed in 7, 12]. Thus, regenerative neurogenesis can be studied in the spatially separate mature/adult (central) retina, which allowed us to established a nitroreductase-metronidazole induced (genetic) ablation model targeted at ablating inhibitory retinal neurons, namely horizontal and amacrine cells at 7 dpf. The efficacy of this injury model was assessed by characterising and comparing its time course, extent, and specificity to a mechanical injury that targeted all retinal neuron types.

**Fig. 1:**
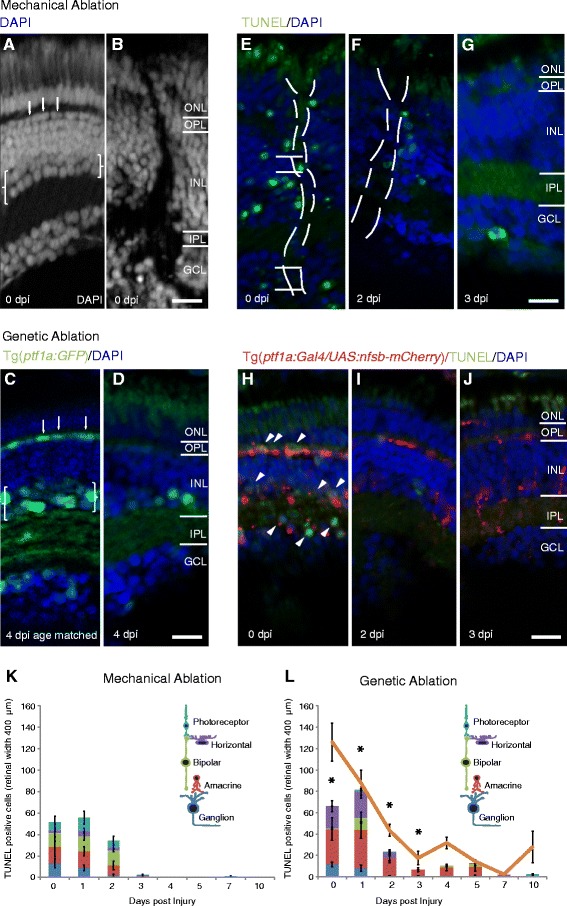
Neuron type specific cell death and comparable regenerative time course in two distinct injury models. **a**-**j** Micrographs of retinal sections after mechanical (**a**, **b**, **e**-**g**) or metronidazole induced genetic ablation in Tg(*ptf1a:Gal4 / UAS:nfsb-mCherry*) (**c**, **d**, **h**-**j**, **a**, **c**) Retinal architecture of the uninjured retina at equivalent ages (**b**, **d**). *Brackets* indicate the amacrine neuron layer (weaker DAPI staining in the inner half of the INL) and *arrows* indicate the horizontal neuron layer (first row of flattened nuclei in the inner nuclear layer – INL). **b**, **d** Retinal architecture of injured retina revealed by DAPI staining shows disruption caused by the needle track immediately after ablation injury (0 dpi), affecting neurons types in each retinal layer (**b**), and loss of horizontal cells and amacrine cells (seen by the reduction in Ptf1a:GFP transgene expression, which specifically labels these two cell types) 4 days after injury, which is a timepoint following the main cell death phase (**d**). **e**-**j** TUNEL labelling at different days post-injury (dpi) in both injury models. TUNEL staining is observed in all retinal layers early after mechanical ablation (**e-g**) and more biased towards horizontal and amacrine cells (*arrowheads* in INL and displaced amacrine cells in GCL) layers among nitroreductase expressing (*red*) cells (**h**-**j**). **k**, **l** Quantification of TUNEL positive cells in the different retinal layers across days post-injury reveals a peak in cell death in the first two days distributed across all retinal layers in the mechanical ablation (**k**) and primarily confined to inhibitory neurons after genetic ablation (**l**) (*n* = 12 larvae per timepoint). *Asterisks* indicate timepoints at which TUNEL labelling was in a significantly higher proportion of inhibitory neurons in the genetic versus mechanical ablation (*p*-value <0.038). **l** Loss of nitroreductase-mCherry positive cells follows the cell death observed in genetic ablation (orange line, *n* = 12 larvae per timepoint). Results are mean ± SEM. ONL: outer nuclear layer; OPL: outer plexiform layer; IPL: inner plexiform layer; GCL: ganglion cell layer; nfsb: Nitroreductase. Scale bar in D (for **a**-**d**) = 50 μm, scale bar in J (for **e**-**j**) = 50 μm

The mechanical needle stick injury is local and we used 6 stabs evenly spaced across the retina to induce wide-spread injury. Immediately after mechanical injury an injury track disrupting all retinal layers was observable (Fig. 1b).

The genetic injury targeted inhibitory neurons using a *ptf1a* promoter [46] to drive the expression of the nitroreductase enzyme, which in turn converts the pro-drug metronidazole into a cytotoxin. By using a transgenic marker of these inhibitory neurons, Tg(*ptf1a:GFP),* the loss of horizontal cell (HC) and amacrine cell (AC) was observed (Fig. 1d). Cell types could also easily be classified by their laminar location, morphology and co-expression of the m-Cherry tag confined to HCs and ACs. The HCs form a single layer of flattened nuclei in the outermost row of the inner nuclear layer and ACs are weaker DAPI-stained neurons in the inner half of the inner nuclear layer (using Tg(*ptf1a:GFP*) the DAPI label distinctions shows only 4.6% false negative (i.e. GFP labelled amacrine cells erroneously assigned to the brighter DAPI labelling in this layer, *n* = 995 cells from 7 larvae).The number of inhibitory neurons was reduced by 51% for amacrine cells (41 ± 2 SEM cells/400 μm retinal width untreated vs. 21 ± 1.5 SEM ​cells/400 μm retinal width post-injury, *n* = 6 and 7 larvae) and 67% for horizontal cells (9 ± 0.775 SEM​ cells/400 μm retinal width untreated vs. 3 ± 0.842 SEM​ cells/400 μm retinal width post-injury, *n* = 6 or 7 larvae).

Cell death was characterised at 0, 1, 2, 3, 4, 5, 7 and 10 days post injury (dpi) using TUNEL labelling (Fig. 1e-j). After mechanical injury, cell death was observed in 52 ± 25.3 SEM cells/400 μm retinal width and peaked at 1 dpi (56 ± 14.8 SEM cells/400 μm retinal width), being almost completely gone by 3 dpi (2.4 ± 0.89 SEM cells/400 μm retinal width) (Fig. 1k). After genetic injury, cell death also peaked at 1 dpi (41 ± 13.8 SEM cells/400 μm retinal width), was reduced by 3 dpi (6.4 ± 0.52 SEM/400 μm retinal width) and almost gone after 5 dpi (Fig. 1l). There was no significant difference between the number of TUNEL labelled cells at any timepoint (student’s *t*-test, *p*-value range 0.09–0.65) except for 4 dpi (student’s t-test, *p*-value = 0.049), suggesting that cell death after genetic injury may continue a little bit longer. The reduction of TUNEL positive and nitroreductase-mCherry (red) labelled cells as time proceeds is due to the clearing by Müller glia, whose processes can be seen to contain the mCherry transgene at 3 dpi (Fig. 1j). Cell death occurred across the retinal layers after mechanical injury (Fig.1k) and primarily in inhibitory layers 81% ± 3.41 SEM ​cells/400 μm retinal width after genetic ablation (arrowheads in Fig. 1j) as compared to 34% ± 6.24 SEM cells/400 μm retinal width post mechanical injury. The proportion of inhibitory neurons lost after genetic injury was significantly higher than the proportion of inhibitory neurons lost after mechanical injury at 0–3 dpi (*p*-value = 0.011 at 0 dpi, 0.0003 at 1 dpi, 0.039 at 2 dpi, 0.004 at 3 dpi) and could not be computed at 4–10 dpi, because there were insufficient TUNEL labelled cells in one or both of the injuries at these timepoints. Consistently, the genetic injury causes a rapid loss of nitroreductase (nfsb) positive cells (Fig. 1l). Thus, differential cell type specific injury with comparable cell death progression was achieved using these two distinct ablation injury models (Fig. 1e-j).

### Progenitor proliferation is comparable in mechanical vs. genetic ablation models

**Fig. 2:**
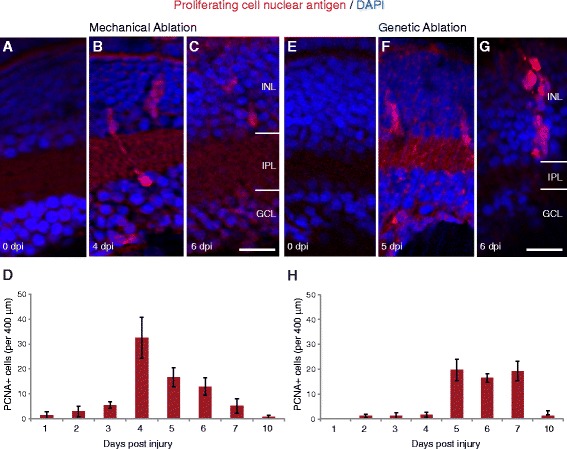
Timing of PCNA labelled proliferation is comparable between injury models. **a**-**c**, **e-g**) Micrographs of retinal sections after mechanical injury (**a**-**c**) and genetic ablation injury (**e**-**g**). Retinal sections stained for PCNA (proliferating cell nuclear antigen, *red*) show cell clusters that span across multiple retinal layers in both injury models (**b**, **f**, **g**). **d**, **h** The graph shows the total number of PCNA cells after mechanical (**d**) and genetic ablation injury (**h**) model, suggesting that broadly, proliferation does not begin until 3–4 dpi and is active for at least three days (*n* = 12 larvae per timepoint per injury model). Results are mean ± SEM. INL: inner nuclear layer; IPL: inner plexiform layer; GCL: ganglion cell layer. Scale bar in G (for **a**–**c**, **e**-**g**) = 50 μm

The temporal stages of progenitor activation and proliferation were compared using immunohistochemical labelling for proliferating cell nuclear antigen (PCNA), a factor expressed during DNA synthesis. In uninjured age-matched controls of the same transgenic lines, there was little proliferation in this central part of the retina (average 0.25–2 PCNA labelled cells/400 μm retinal width, *n* = 50 retinas). PCNA positive cell clusters suggestive of clones arising from individual cells were observed after mechanical injury (Fig. 2b), with a peak between 4 and 6 dpi (Fig. 2d, 4 dpi: 32.8 ± 8.33 SEM cells/400 μm retinal width; 5 dpi: 16.8 ± 3.82 SEM cells/400 μm retinal width; 6 dpi: 12.8 ± 3.51 SEM cells/400 μm retinal width) and after genetic injury (Fig. 2f), with a peak between 5 and 7 dpi (Fig. 2h; 5 dpi: 19.6 ± 4.34 SEM cells/400 μm retinal width; 6 dpi: 16.4 ± 1.68 SEM cells/400 μm retinal width; 7 dpi: 18.8 ± 3.87 SEM cells/400 μm retinal width). The slightly earlier proliferation after mechanical ablation may be due to the acute cell damage and “death” signal being present immediately, in contrast to the genetic model, which relies on conversion of prodrug, accumulation of toxin, and robust activation of apoptotic pathways. Nonetheless, the period of peak proliferation occurs primarily over a 2-day window at a broadly similar time following either injury.

### Regenerating proliferative cells arise from Müller glia

The predominant regenerative cell source after large injuries in the zebrafish retina is the Müller glia [1–3, 11, 14, 32, 47]. A GFP reporter protein was used to label Müller glia Tg(*gfap:GFP*) in addition to co-labelling with proliferation markers to confirm that progenitors originated from Müller Glia .

**Fig. 3:**
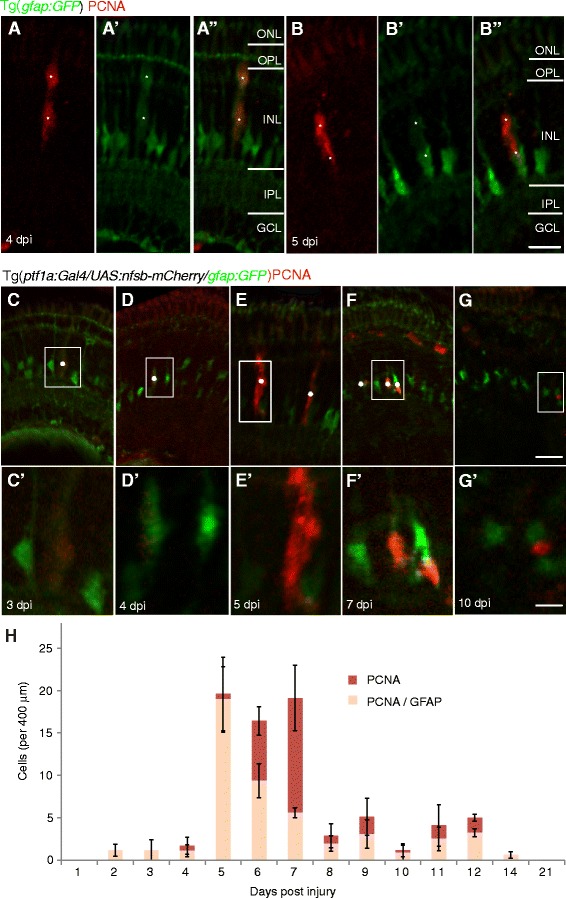
Progenitors and clones arise from Müller glia. **a**-**g** Micrographs of retinal sections of Tg(*gfap:GFP*) lines, with Müller glia cells (*green*) stained for PCNA (proliferating cell nuclear antigen red). As previously published, the needle stick injury causes proliferation in Müller glia (**a**). In our newly established genetic injury, PCNA labelled proliferation was also in Müller glia (**b**). **c**-**h**) A detailed time series and quantification (**h**) shows the peak proliferative stage during 5–7 days post-injury (dpi) (*n* = 12 larvae per timepoints 1–10 dpi, *n* = 8 larvae per timepoints 11–14, *n* = 6 larvae at 21 dpi). Proliferative cells in the first 5 dpi also almost exclusively co-labelled with progressively weaker GFAP:GFP, after which time there were also many proliferative cells that no longer expressed detectable GFAP:GFP. White insets (**c**’-**g**’) show higher power magnification of boxed region indicated in **c**-**g**. Results are mean ± SEM. ONL: outer nuclear layer; OPL: outer plexiform layer; INL: inner nuclear layer; IPL: inner plexiform layer; GCL: ganglion cell layer. Scale bar B (for **a**-**b**) = 50 μm, scale bar in G (for **c**-**g**) = 50 μm, scale bar G’ (for **c**’-**g**’) = 200 μm

Mechanical injury was conducted in Tg(*gfap:GFP*) and stained for PCNA at 3, 4, 5, 6 & 7 dpi (Fig. 3a-b) confirming previous studies showing that proliferating cells arose from GFAP labelled Müller glia cells. Similarly, genetic injury conducted in Tg(*ptf1a:Gal4/UAS:nfsb-mCherry/gfap:GFP*) transgenics and stained for PCNA at 1, 2, 3, 4, 5, 6, 7, 8, 9, 10, 11, 12, 14, 21 dpi (Fig. 3c-h) also revealed that Müller glia are the main proliferative cell source following this novel injury paradigm (Fig. 3c-g). At 5 dpi, 97% of all PCNA cells were co-labelled with Gfap:GFP, though most of the co-labelled glia showed a reduction of GFP level as compared to neighbouring non-proliferative glia (Fig. 3a, b). At subsequent days, PCNA labelled cells co-labelled with GFAP:GFP reduced to 57% at 6 dpi and 29% at 7 dpi consistent with de-differentiation (downregulation of GFAP and other glial markers) in these activated cells. This confirms the primary cell source of progenitors in both injury models was the Müller glia cell population.

### The environment directs cell type specific regeneration at early stages

**Fig. 4:**
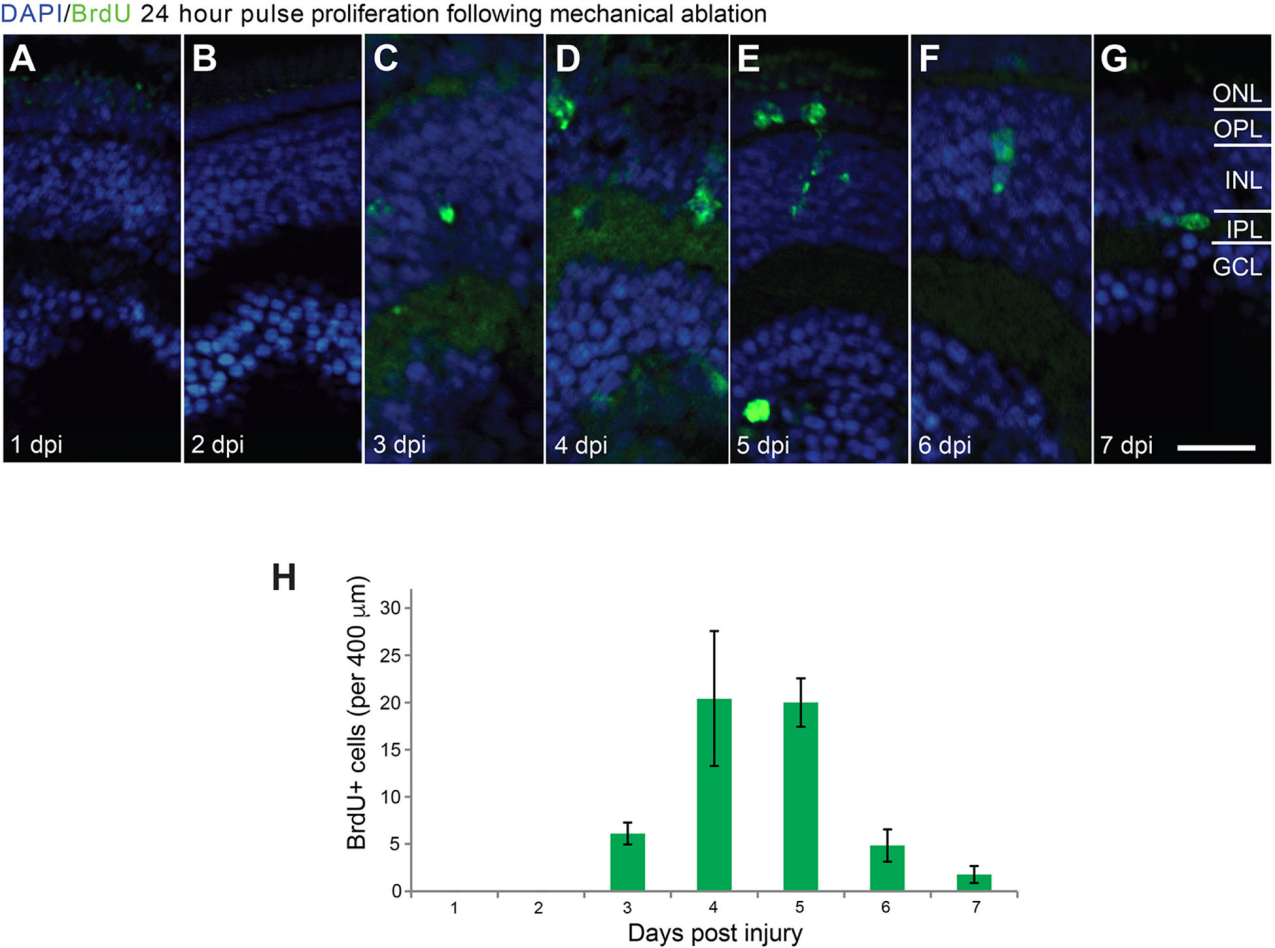
Proliferation time course measured with 24 h pulse BrdU incorporation is comparable to PCNA time course. **a-g**) Micrographs of retinal sections after mechanical injury stained with DAPI (*blue*) and for BrdU (*green*). **a-g**) BrdU positive cell clusters were observed between 3 to 7 days post-injury (dpi) with cells across multiple retinal layers. **h** The graph shows that BrdU positive cells were most abundant within a 2–3 day time period (*n* = 12 larvae). Results are mean ± SEM. *Scale bar* G (for **a-g**) = 50 μm

In order to determine fate specification during regeneration, we performed prolonged 5-bromo-2′-deoxyuridine (BrdU) labelling across the peak proliferative phase following injury. Because BrdU incorporation and PCNA cell cycle snapshot may differ, we utilised the mechanical injury to compare the proliferative phase identified with PCNA labelling using daily 24 h BrdU pulses . Highest BrdU incorporation occurred at 4 dpi (20.4 ± 0.38 SEM cells/400 μm retinal width) with a reduction by 7 dpi (2 ± 1.07 SEM cells/400 μm retinal width) (Fig. 4a-g), and matched the time course identified by PCNA staining.

**Fig. 5:**
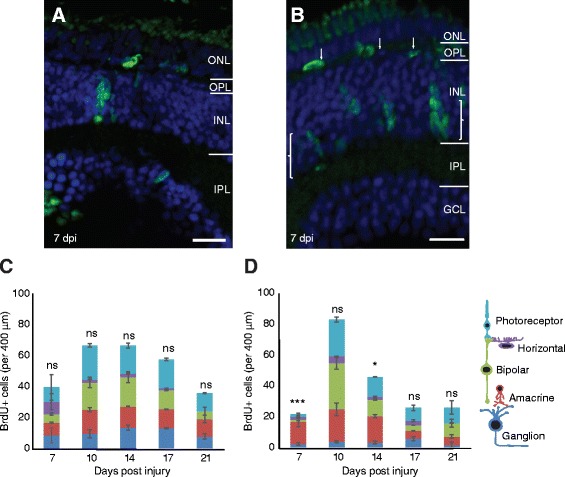
Prolonged BrdU exposure reveals cell type specific replacement. **a**, **b** Micrographs of mechanical and genetic ablated juveniles in prolonged BrdU exposure between 3 and 7 dpi. Retinal lamination has recovered by this timepoint with horizontal cells (*arrows*) and amacrine cell layer (*brackets*) re-establishing after genetic ablation. **c**, **d** Graphs indicating the total number of BrdU cells in each retinal layer across 5 time points observed in the mechanical (**c**) and genetic (**d**) ablation injury models. Statistics indicate comparison of the proportion of inhibitory neurons compared to age-matched uninjured control composition. After genetic ablation the vast majority of proliferative cells at 7 dpi are confined to the inhibitory layers, most notably the amacrine layer (*** *p*-value = 2.2 × 10^−7^ compared to WT proportion). In both injuries, the total number of cells per layer increases after 7 dpi and decreases by 14 dpi (genetic) and 17 dpi (mechanical) (*n* = 12 larvae at 7 and 10 dpi, 8 larvae at 14 dpi and 6 larvae at 17 and 21 dpi). Ns: not significant (*p*-value >0.05), * *p*-value = 0.004. Results are mean ± SEM. ONL: outer nuclear layer; OPL: outer plexiform layer; INL: inner nuclear layer; IPL: inner plexiform layer; GCL: ganglion cell layer; *Scale bars* = 50 μm

**Fig. 6:**
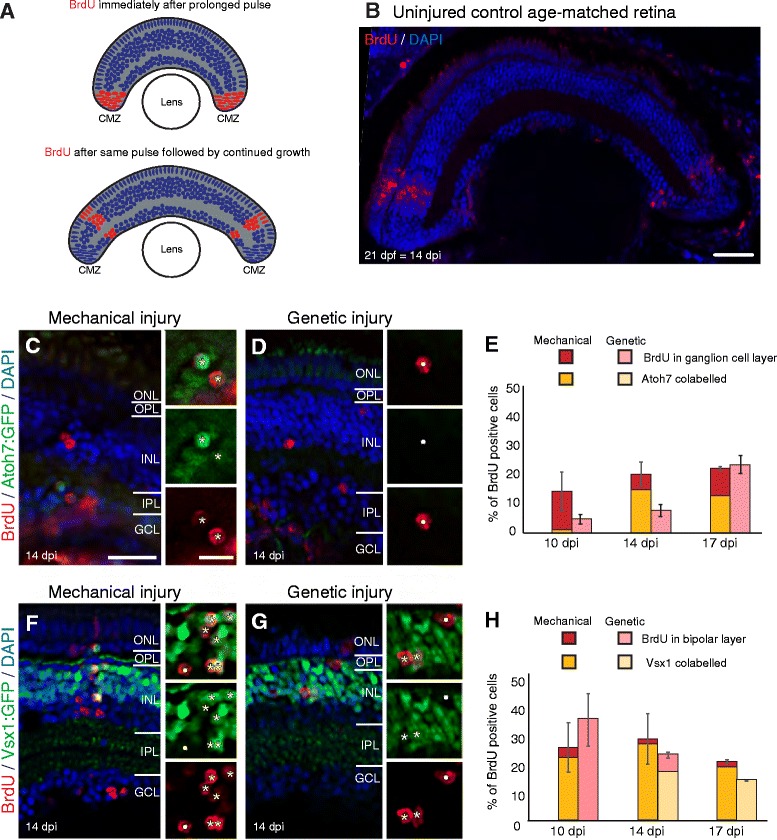
Fate determinant expression during regeneration does not recapitulate developmental sequence after different injuries. **a**, **b**) In uninjured control, a prolonged BrdU pulse labels neurons in the peripheral ciliary margin zone, which results in a stripe of BrdU positive cells after BrdU withdrawal, as BrdU negative cells continue to be added from the ciliary margin. This BrdU stripe is observed in micrographs from control (**b**). There are no BrdU cells in the mature retina found more centrally. **c**-**h**) Using prolonged exposure, BrdU labelled cells observed in this central mature retina region reflects regeneration. Micrographs show retinal sections from 14 days post-injury (dpi). The proportion of BrdU positive GCL cells after mechanical injury (*n* = 17 larvae - 10 dpi, 9 larvae −14 dpi, 12 larvae - 17 dpi) is higher compared to genetic injury (*n* = 8 larvae - 10 dpi, 15 larvae - 14 dpi, 19 larvae - 17 dpi) at 10 and 14 dpi. The firstborn ganglion cell marker Tg(*atoh7:GFP*) shows more co-labelling after mechanical injury. A large proportion of BrdU positive labelled cells in the bipolar layer (outer half of INL) show high expression of Tg(*vsx1:GFP*) indicative of bipolar differentiation (last born during development) after both injuries, starting earlier after mechanical (*n* = 13 larvae - 10 dpi, 24 larvae - 14 dpi, 21 larvae - 17 dpi) than genetic (*n* = 14 larvae - 10 dpi, 21 larvae - 14 dpi, 11 larvae - 17 dpi) injury. For both injuries, strongly labelled Vsx1 cells are observed prior to strongly labelled Atoh7 GCL cells. Results are mean ± SEM ONL: outer nuclear layer; OPL: outer plexiform layer; INL: inner nuclear layer; IPL: inner plexiform layer; GCL: ganglion cell layer. Scale bar B = 100 μm, scale bar C (for **c**, **d**, **f**, **g**) = 50 μm, scale bar in insets C (for *insets* in **c**, **d**, **f**, **g**) = 20 μm

Thus, zebrafish were treated after injury with a prolonged BrdU pulse by incubation in BrdU overnight (16 h) and daily from 3 to 7 dpi to encompass the main proliferative stage (Fig. 5a, b). Leaving larvae in BrdU for the entire period unexpectedly resulted in less BrdU labelled cells, and the zebrafish started to show detrimental health, suggesting extensive exposure may have toxic side effects (data not shown). Because BrdU labelled cells can retain the label for additional cell cycles (before being diluted out), this paradigm should label the vast majority, if not all of the newly generated proliferating cells. Control uninjured age-matched tissue labelled only few cells (average 0–0.6 cells/400 μm retinal width, *n* = 7–9 larvae, Fig. 6a, b). The prolonged BrdU pulse labelled 47 cells ±14.88 SEM cells/400 μm retinal width 7 dpi after mechanical injury and 68 cells ±11.66 SEM cells/400 μm retinal width 7 dpi after genetic injury (Fig. 5a, b). Following BrdU exposure withdrawal, the BrdU cell number continued to increase, suggesting that the labelled population may continue dividing.

The proportion of BrdU labelled cells was compared to the normal distribution of retinal neurons in a WT uninjured control, where we quantified 12.5% photoreceptors, 6.4% horizontal cells, 30.4% bipolar cells, 15.5% amacrine cells, 28% displaced amacrine cells and ganglion cells (DAPI labelled Tg(*ptf1a:GFP*) retinas, *n* = 795 cells from 5 larvae). In particular, we quantified the proportion of BrdU cells that gave rise to the inhibitory neurons that were particularly targeted with the genetic, but not mechanical injury. After mechanical injury (Fig. 5c) BrdU positive cells were found in all retinal layers at all time points. There was no significant difference in the proportion of labelled cells found in inhibitory layer at any of the time points (student’s *t*-test, *p*-value ranged from 0.10 to 0.74).

**Fig. 7:**
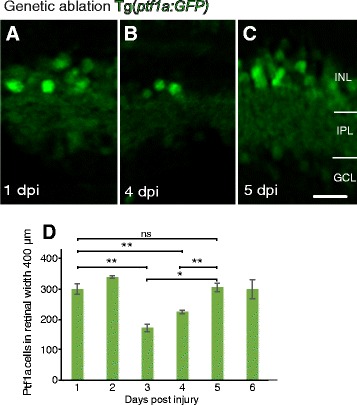
Following genetic ablation, new horizontal and amacrine cells can be observed prior to the proliferative wave. **a**-**c**) Micrographs of retinal sections in Tg(*ptf1a:GFP*) larvae at different days post injury (dpi). **d** Quantification shows an initial reduction and subsequent increase in the number of Ptf1a:GFP labelled inhibitory neurons. At 3 and 4 dpi, the number is significantly lower (* *p*-value = 0.018, ** *p*-value ≤0.01) compared to 1 dpi (baseline) or 5 dpi (regenerated), which are not significantly different from each other (*p*-value = 0.50). Ns: not significant (*p*-value >0.05). Results are mean ± SEM. INL: inner nuclear layer; IPL: inner plexiform layer; GCL: ganglion cell layer. Scale bar C = 50 μm

After genetic injury (Fig. 5d) at 7 dpi, BrdU positive cells were mainly distributed in the amacrine and horizontal layers (75% ± 4.8% SEM), which was significantly different from the WT distribution of inhibitory cells (student’s t-test, *p*-value = 2.2 × 10^−7^). From 10 dpi onwards, proliferating cells were also distributed across other neural layers and showing less pronounced, but still significantly higher representation of inhibitory neurons at 14 dpi (*p*-value = 0.004), but not 10 dpi (*p*-value = 0.11) or 17 dpi (*p*-value = 0.21). By 7 dpi, the retinal laminar architecture started to recover. Quantification of horizontal and amacrine cells following genetic ablation using Tg(*ptf1a:GFP*) revealed a reduction in GFP positive horizontal and amacrine that was significantly different from 1 dpi (student’s *t*-test, *p*-values = 0.01 (3 dpi) and 0.01 (4 dpi), and 5 dpi (student’s *t*-test, *p*-values = 0.018 (3 dpi) and 0.007 (4 dpi). By 5 dpi, there was no significant difference compared to 1 dpi (student’s *t*-test, *p*-value = 0.50) (Fig. 7), suggesting that the initial wave of biased cell regeneration had re-established cellular proportions. Thus, the bias towards specific cell types might remain a dynamic process that continues to adapt to the changing environmental signals as regeneration progresses.

### Sequence of fate specification gene expression in proliferative regenerated neurons is distinct from development

During developmental neurogenesis, retinal neuron types are born in a highly conserved histogenic order [48–53]. This process is controlled by the sequential intrinsic expression of fate specification factors. Extrinsic influences can bias or direct fate specification during development at least in part by affecting the timing of such intrinsic fate specification factors [20–25, 54]. Because our injury models result in an initial fate bias, we compared the expression of transcription factors that indicate earliest born (ganglion cell) and latest born (bipolar cell) neurons to assess whether the same sequential gene expression occurs during regeneration.

Both injuries were conducted in transgenic lines Tg(*atoh7:GFP*) (Fig. 6c–e) and Tg(*vsx1:GFP*) (Fig. 6f–h). The bHLH atonal homolog 7 (Atoh7) specifies earliest born ganglion cell fate [20, 55, 56] and the visual homeobox transcription factor 1 (Vsx1) is expressed at medium levels in retinal progenitors and upregulated strongly in differentiating last born bipolar cells [57]. Detection of these transgenes allows us to identify neuron cell specification at an early differentiation stage. Using the prolonged BrdU pulse, we compared the time course of gene expression versus retinal layer distribution of BrdU positive cells at 7, 10, 14 and 17 dpi.

In Tg(*atoh7:GFP*) mechanically injury model, 23% (14.8 ± 8.16 SEM cells/400 μm retinal width) of all BrdU positive cells were located in the ganglion cell layer by 14 dpi, and 75% (11 ± 5.69 SEM ​cells/400 μm retinal width) of these co-labelled with Atoh7:GFP. Similar results were observed at 17 dpi. Thus, cells within the ganglion cell layer migrated appropriately and started differentiating at least at 14 dpi. Similarly, 24% of BrdU positive cells were also located in the ganglion cell layer in the genetic ablated Tg(*atoh7:GFP*) cohort by 17 dpi. However, none of these cells expressed Atoh7:GFP at any stage of our analysis, although Atoh7 expression was turned on at 17 dpi in both injuries in BrdU positive cells in the inner half of the inner nuclear layer (20% in mechanical injury; 15% in genetic ablation injury), which is occupied by amacrine cells, a subset of which also arise from this lineage [21, 58]. Thus, after genetic injury, where inhibitory neurons are regenerated first, the generation of ganglion cells and differentiation seems to be delayed relative to the mechanical injury model.

In development, Vsx1 is strongly upregulated in cells as they differentiate into the last born bipolar retinal cell type, which is easily distinguished from the weaker expression in progenitors [57]. In our mechanical injury model, 78% of all BrdU cells in the bipolar layer (17 ± 8.45 SEM cells/400 μm retinal width) expressed strong Vsx1:GFP signal already at 10 dpi. In the genetic ablation injury model, 73% of all BrdU cells in the bipolar layer (6.8 ± 0.96 SEM BrdU positive cells/400 μm retinal) were co-labelled with Vsx1:GFP at 14 dpi. Vsx1:GFP expression was strongly maintained in all BrdU positive cells in the appropriate retinal bipolar layer at the later stages in mechanical (95%, 14 &17 dpi) and genetic (100%, 17 dpi) ablation models. Thus, as is the case with Atoh7, differentiating Vsx1:GFP expressing bipolar cells are also only generated at a later time point in the genetic ablation model, in which inhibitory neurons are preferentially regenerated first.

These results also indicate that regenerated cells migrate to the their correct laminar location within the retina according to their fate specification. Additionally, after both injuries the timing of expression of Atoh7 (starting 14 dpi in mechanical and 17 dpi in genetic injury) compared to Vsx1 (at 10 dpi in mechanical and 14 dpi in genetic injury) seems to be reversed compared to development. In development Atoh7 is first upregulated at 28 hpf to start generating ganglion cells [20] and Vsx1 is only upregulated at 35 hpf to start generating bipolar cells [57]. Thus, the regeneration of different neuron types may not strictly follow the stereotypical processes observed during development.

## Discussion

The vertebrate neural retina allows us to assess regenerative processes in a well-characterised and highly organised neural tissue. While signalling pathways involved in retinal regeneration are being identified and expanded, how progenitor cells use these pathways to make fate decisions remains unclear.

Little is known about how pre-programmed versus adjustable fate choices operate in vivo and how the injury environment influences regenerative outcomes, such as determining the fate choice of progenitors to repopulate lost neurons. While the number of each cell type seems to be controlled independently [59] there exists plasticity within the CNS (e.g. neurite arbor size) to compensate by varying in cell type produced [60]. During development, such environmental contributions were described in fish [20, 21, 25, 54] and Xenopus [23, 24], showing that progenitors can be biased towards generating more of the missing subtypes.

There is mounting evidence that regenerating neurons use extrinsic feedback to drive preferential fate specification bias in zebrafish [33, 35, 36]. In our study, we identify a key relative early time point within the first week post injury, where fate specification is biased strongly towards the ablated cell type. Further, our data shows that feedback is dynamic, as progenitors adjust their fate bias as the cell type proportions are restored throughout this regenerative process. Thus, the strong fate bias found early in regeneration reduces as the environment reaches appropriate neural composition. This means that extrinsic feedback is utilised throughout the regenerating period, not only present at the initial stem cell activation phase. Thus, our data supports the hypothesis that intrinsic highly conserved mechanisms such as sequential fate specification factors may be suppressed during regeneration.

Both of our injuries resulted in regenerative responses comparable in timing and extent of cell death and Müller glia driven proliferation. This was important to establish given different paradigms can lead to different regenerative responses [8–10, 47]. Preliminary experiments using 1 or 2 stabs (data not shown) showed a clustered distribution of fewer proliferating cells, consistent with signals triggering regeneration being spatially limited. However, 6 stabs were found to be enough to trigger a proliferative response that was similar in cell number and spatial distribution to that observed in the genetic model. Using prolonged BrdU pulse (3–7 dpi) to label the bulk of regenerating cells, we quantified the differentiation of ablated and non-ablated cell types as regeneration progressed whilst tracking the recovery of retinal architecture and neural proportions. While the prolonged BrdU paradigm consists of 16-h on/8-h off exposure for the benefit of animal health, BrdU can be detected through a few divisions after removal of BrdU. This is consistent with the observation that the number of BrdU cells initially increased beyond 7 dpi (when BrdU exposure was stopped).

At later stages after both injuries, the number of BrdU labelled cells unexpectedly declined and more so after genetic injury. This could be due to newly generated cells undergoing apoptosis, which may be a real biological phenomenon (e.g. cells that do not integrate into circuits) or an artefact of the experimental approach (e.g. cell toxicity due to the prolonged BrdU pulse). Alternatively, proliferation may continue or increase causing a dilution of the BrdU signal.

The mechanical injury resulted in unbiased regeneration of all neuron types. In contrast, genetic ablation resulted in the specific regeneration of the targeted inhibitory neuron types, particularly at early stages of regeneration (7 dpi). Hence, in the genetic ablation model extrinsic fate strongly influenced neural regeneration in line with fate biases and layer selective migration observed in previous zebrafish studies [31–34]. Since our experiments are conducted in young larvae to minimise frequency of metronidazole treatment, the regenerative time course is possibly accelerated compared to adult models [36]. By combining data obtained from labelling different cohorts of proliferative cells [36] with our current work of labelling all cohorts and assessing progression of fate specification throughout the differentiation stages, we propose three key stages of fate determination. Initially, proliferative progenitors may be deployed to all retinal layers in an intrinsic multipotent fashion [36], followed by a second proliferative expansion phase driven by extrinsic feedback to initially replace only the affected neuron types. Finally, at later stages (10 dpi onwards in our genetic injury), proliferative cells also differentiate into non-ablated cell types. Because an initially fate biased regeneration gradually restores normal cell type proportion, the extrinsic feedback will similarly become less fate biased. Thus, the observation that newly generated cells differentiate into all neuron types at later stages suggest progenitors continue to adapt to this new cellular environment to give rise to all retinal cell types. This last phase may still be primarily extrinsically driven rather than requiring a switch back towards an intrinsic pre-programmed mechanism.

The specification of non-ablated neurons at later stages, may indicate an excess number of neurons being regenerated. However, even the peak number of BrdU labelled cells following the prolonged pulse only accounts for half of the number of observed TUNEL positive cells, with TUNEL itself representing only a snapshot of dying cells. Since no striking expansion of layers containing non-ablated cell population was observed, massive overproduction does not seem to be occurring. Nonetheless, it would be an interesting to study newly made non-ablated cell types and assess, how their generation influences overall proportions, neural circuitry and whether appropriate pruning off via cell death occurs.

An intriguing observation following genetic injury is the rapid restoration of retinal inhibitory cells (Fig. 7) by 7 dpi. This occurred despite the number of proliferative cells being too low to account for such extensive regeneration of these ablated neuron types. Therefore, this raises the possibility that restoration of these inhibitory neuron layers may also include non-proliferative contributions from alternate cell sources, which requires further investigation.

Our results show evidence of disruption to the developmental histogenic processes [48, 50, 52, 53, 61]. This was demonstrated by a failure to recapitulate the birth order of last born bipolar cells and first born ganglion cells as both cells expressed transgenes simultaneously. This adds to the evidence of flexibility in cell regeneration processes to shift from the highly co-ordinated gene expression during development towards a more environmental driven process involving more feedback and less rigid intrinsically timed fate progression. A comprehensive fine-scale time course including markers for each fate and clonal analysis would confirm this.

Proliferative cells found in the INL may represent different cell populations. At early regenerative stages, BrdU could be labelling activated Müller glia and early glia derived progenitors, which usually reside in the INL. At intermediate regenerative stages, BrdU labelled cell within the INL could represent progenitor cells undergoing interkinetic nuclear migration (IKNM) cells [62], which occurs during development [63–65], or differentiating cells undergoing their final laminar migration. At late stages, at least after 10 dpi, the co-labelling with the bipolar Vsx1:GFP transgene shows high correlation, suggesting that BrdU labelled cells found in the INL at this stage, are differentiating or mature postmigratory neurons.

## Conclusions

We show that the environment after an injury can efficiently and accurately drive neurogenesis, a field that has been previously dominated by contributions of intrinsic gene control. This may be a stronger driver and independent from developmental mechanisms. This data supports alternative approaches to using existing methods that currently direct stem cells in vitro towards a cell specific fate for transplantation therapies. Since visual and other neurodegenerative disorders usually only affect specific neural types, the innate environment may be able to direct the progenitor fate biases. Retinal progenitors introduced early into a host environment may be able to use the extrinsic feedback and existing scaffold to restore correct neuron type proportions. Early integration could also assist other differentiation steps such as migration, pathfinding and re-establishment of neural circuit, that depend on such environmental signals during development. While the processes described during development form an important starting point for our understanding of regeneration, further comparative studies are needed translate such knowledge towards the human clinical setting [16, 66].

## Abbreviations

Atoh7: Atonal homolog 7
BrdU: 5-bromo-2′-deoxyuridine
DAPI: 4′,6-diamidino-2-phenylindole
dpf: Days postfertilisation
dpi: Days post-injury
hpf: Hours postfertilisation
nfsb: Nitroreductase
PBS: Phosphate buffered saline
PCNA: Proliferating cell nuclear antigen
PFA: Paraformaldehyde
Ptf1a: Pancreas transcription factor 1 a
sem: Standard error of the mean
TUNEL: Terminal deoxynucleotidyl transferase dUTP nick end labelling
UAS: Upstream activating sequence
Vsx1: Visual homeobox transcription factor 1
WT: Wildtype
